# Preclinical Risk Assessment of Plant Lectins with Pharmacological Applications: A Narrative Review

**DOI:** 10.3390/molecules31010055

**Published:** 2025-12-23

**Authors:** Amanda de Oliveira Marinho, Maria Nívea Bezerra da Silva, Suéllen Pedrosa da Silva, Isabella Coimbra Vila Nova, Jainaldo Alves da Costa, Patrícia Maria Guedes Paiva, Lidiane Pereira de Albuquerque, Emmanuel Viana Pontual, Leydianne Leite de Siqueira Patriota, Thiago Henrique Napoleão

**Affiliations:** 1 Departamento de Bioquímica, Centro de Biociências, Universidade Federal de Pernambuco, Recife 50670-901, Brazil; amanda.marinho@ufpe.br (A.d.O.M.); maria.nivea@ufpe.br (M.N.B.d.S.); suellen.pedrosa@ufpe.br (S.P.d.S.); isabella.coimbra@ufpe.br (I.C.V.N.); jainaldo.costa@ufpe.br (J.A.d.C.); patricia.paiva@ufpe.br (P.M.G.P.); leydianne.patriota@ufpe.br (L.L.d.S.P.); 2 Departamento de Bioquímica e Farmacologia, Centro de Ciências da Saúde, Universidade Federal do Piauí, Teresina 64001-020, Brazil; lidianealbuquerque@ufpi.edu.br; 3 Departamento de Morfologia e Fisiologia Animal, Universidade Federal Rural de Pernambuco, Recife 52171-900, Brazil; emmanuel.pontual@ufrpe.br

**Keywords:** safety assessment, lectin, cytotoxicity

## Abstract

Plants have been used for medicinal purposes both intuitively and based on traditional knowledge for centuries. Recently, however, there has been a significant increase in research focused on medicinal plants to meet the growing demands of the pharmaceutical industry. As a result, it has become essential to evaluate the safety of natural products for human use. This review examines in vitro and in vivo toxicity studies of lectins, a class of plant proteins with pharmacological applications. The reviewed data indicate that many of these proteins do not appear to be toxic to human and animal cells, nor when administered to rodents through oral, intraperitoneal, or intravenous routes. However, some lectins have shown toxicity under certain conditions, such as depending on the administration route, dose, and treatment duration. These adverse effects may include behavioral changes, antinutritional effects, hepatotoxicity, nephrotoxicity, pancreatic hypertrophy, allergic reactions, and even death. Therefore, it is crucial to prioritize toxicological studies to ensure the safety of these plant proteins as potential drug candidates in the future.

## 1. Introduction

Traditional medicine relies on natural products, including plant-based preparations, which play a significant role in the treatment and management of diseases in humans and animals. This approach has been widely adopted to meet the basic health needs of populations, often serving as a complement and/or alternative to conventional treatments [1].

Since ancient times, plants have been used for medicinal purposes. However, this use was primarily based on intuition and popular experience, as there was limited knowledge about their chemical composition and pharmacological mechanisms. Over time, scientific research has validated the use of medicinal plants, identifying their active ingredients, elucidating their mechanisms of action, and providing critical insights into safe dosage limits to minimize potential adverse effects [2]. Currently, natural products and long-standing traditional knowledge underpin about 40% of pharmaceuticals, such as aspirin, artemisinin, and drugs used for childhood cancer [3], and isolated compounds or their structural analogs are widely used in clinical practice [4].

Despite their significant relevance in the medical field, studies have shown that some plant compounds can cause unwanted and adverse reactions, including carcinogenicity, teratogenicity, and even lethal effects [5,6,7]. Therefore, it is essential to conduct thorough testing to evaluate the efficacy and safety of these compounds before they are formulated and marketed as herbal medicines. In addition, herbal medicines are difficult to standardize because plant materials vary widely in their chemical composition depending on species, growing conditions, and harvesting methods, which can affect both safety and effectiveness. Ensuring batch-to-batch consistency requires reliable quality-control testing using validated analytical tools to identify and measure active compounds. Clinical trials are also challenging because differences in dosage, treatment duration, and patient response can lead to either toxicity or poor effectiveness. Using updated clinical-trial guidelines can help standardize dosing, improve quality, and support the safe and cost-effective integration of traditional plant-based therapies into healthcare systems [8,9].

In response to adverse environmental conditions or attacks by aggressors, plants employ various defense strategies. These strategies help them cope with abiotic stresses, such as temperature fluctuations, water scarcity, or exposure to chemical agents, in addition to biotic stresses from phytopathogenic agents like fungi, bacteria, viruses, and nematodes, as well as herbivorous and predatory animals [10,11,12]. Molecules such as lectins are produced by plants to serve, among other functions, as defense mechanisms. As a result, these proteins have garnered significant interest in Biotechnology and Medicine.

Lectins are proteins that specifically and reversibly recognize carbohydrates and are found in microorganisms, plants, and animals [13]. These proteins exhibit a wide range of biological activities, which have sparked significant interest in biomedical research for their potential use in the diagnosis and treatment of diseases and infections [14,15]. Previous reviews have examined the toxicity of plant lectins, mainly focusing on their antinutritional effects following ingestion, summarizing evidence that lectins can induce harmful reactions in the gastrointestinal tract (damage to epithelial cells and luminal membranes, disruption of nutrient digestion and absorption, disturbance of gut immunological homeostasis, and interference with the microbiota) and systemic effects (metabolic disturbances and organ enlargement or atrophy) [16,17]. In addition, some lectins from pulses are known to cause acute symptoms such as nausea, vomiting, and diarrhea when consumed [18]. The toxicity of lectins has also been reviewed in the broader context of plant toxic proteins, with particular emphasis on their antinutritional properties [19].

The range of pharmacological activities attributed to lectins has expanded considerably over the past few decades. In parallel, concerns regarding their safety have broadened beyond issues related solely to ingestion and antinutritional effects. In this context, the present work sought to develop a narrative review of studies investigating the toxicity of lectins with demonstrated pharmacological potential, considering multiple routes of administration. The goal was to identify key safety concerns and potential health risks that may influence or limit their biomedical application. By consolidating current evidence, this review aims to provide guidance for future research and support the safe and informed development of lectin-based therapeutics.

## 2. Lectins

**Figure 1:**
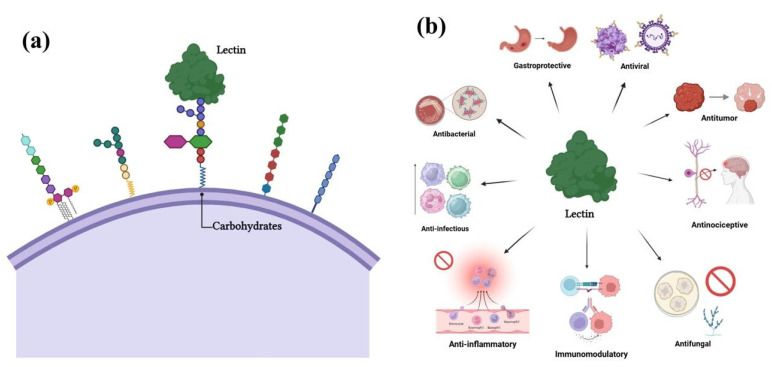
Lectins and their biological activities. (**a**) Schematic representation of lectin-carbohydrate interactions on the cell surface. The binding of lectins to cell-surface carbohydrates plays a crucial role in regulating cellular responses. (**b**) Diverse biological activities associated with lectins. This broad spectrum of activities highlights the potential of lectins as therapeutic agents for treating infections, inflammation, cancer, and other health conditions. Created using BioRender.

Lectins are a group of proteins that specifically and reversibly recognize and bind to carbohydrates (Figure 1a). These carbohydrates can be free or found in the form of glycoconjugates, such as glycoproteins and glycolipids, or as polysaccharides. The structure of the carbohydrate recognition domains (CRD) present in lectins plays a crucial role in determining the specificity of the interaction with carbohydrates. This interaction is facilitated by van der Waals forces, hydrophobic interactions, and hydrogen bonds [20].

Lectins share the ability to bind carbohydrates, but their molecular structures are highly diverse, as are their biochemical and biophysical characteristics. Moreover, no direct correlation has been established between the structural features of the CRD and binding specificity. Classifications based on domain architecture and lectin motifs have been proposed, resulting in the definition of 12 lectin families [21]. However, the three-dimensional structures of many lectins have yet to be resolved.

Based on the relationship between structure and the number and type of CRDs, lectins have been classified into merolectins (which contain only one CRD), hololectins (which contain two or more CRDs), superlectins (which also contain two or more CRDs but with different binding specificities), and chimerolectins (which have one CRD linked to one or more domains with other functions). It is also now recognized that many lectins preferentially interact with complex carbohydrates rather than with monosaccharides [20,21].

Lectins are widely distributed in nature, occurring in plants, animals, and microorganisms. In plants, lectins serve a protective role against environmental stress and attacks from predators and pathogens [20]. These proteins can be isolated from various plant parts, including barks, seeds, leaves, flowers, roots, rhizomes, and fruits. Plant-derived lectins represent a highly heterogeneous group, with evolutionary differences in biochemical and physicochemical properties, molecular structure, and carbohydrate-binding specificity [15,20].

Carbohydrates play a key role in several biological processes, including cell interaction, communication, and adhesion. Lectins, with their ability to recognize carbohydrates, are capable of decoding critical information in many of these processes. As a result, a wide variety of biological responses mediated by lectins have been described (Figure 1b). Lectins can exhibit inhibitory, microbicidal, and antibiofilm activities against pathogenic microorganisms [15,22], as well as interfere with viral attachment, entry, and replication [23]. Antitumor activity of lectins has also been reported, with these proteins able to induce cancer cell death and inhibit angiogenesis around tumors [24,25]. Lectins exert anti-inflammatory effects by modulating the immune response, reducing inflammatory mediators (such as cytokines, NO, and prostaglandins), and regulating the activation of immune cells, including macrophages, neutrophils, and lymphocytes [26]. Their antinociceptive effects have been associated with modulation of immune–inflammatory pathways and neuronal signaling, including interactions with different types of nociceptors [27]. In addition, the neuromodulatory properties of lectins have been described in several studies investigating the anxiolytic and antidepressant effects of these proteins [28].

**Figure 2:**
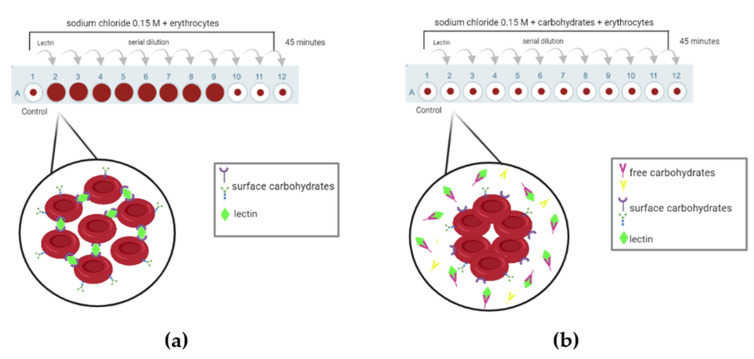
Hemagglutinating activity test for lectin detection. (**a**) Schematic representation of the hemagglutinating activity test, where lectins bind to carbohydrates present on the erythrocyte surface, forming a hemagglutination network. (**b**) In the hemagglutinating activity inhibition test, carbohydrates or glycoconjugates are added to the solution. The lectin present in the sample specifically interacts with these carbohydrates, resulting in the deposition of erythrocytes at the bottom of the well. Created using BioRender.

Unlike merolectins, the hololectins and superlectins are able to agglutinate cells because they possess at least two CRDs, a characteristic that is commonly used for their rapid detection. One of the most commonly used methods for detecting the presence of lectins is the hemagglutination assay. In this test, if a lectin is present in the sample, it binds to sugars on the surface of erythrocytes, forming a hemagglutination network. If this interaction does not occur, the erythrocytes settle at the bottom of the plate (Figure 2a). To confirm that the hemagglutination network was indeed caused by a lectin, a hemagglutination inhibition assay is performed (Figure 2b). In this assay, free carbohydrates are added to the solution, which can bind to the lectin, preventing it from interacting with the carbohydrates on the surface of the erythrocytes. In turn, merolectins can be generally detected and purified by affinity methods.

The purification of lectins does not follow a universal protocol due to the particularities of the source materials and the wide diversity of properties exhibited by these proteins. Consequently, the procedure may involve clarification and concentration steps, and both the number and type of chromatographic steps can vary. Affinity chromatography is an important tool for purifying many lectins. However, for industrial-scale production and widespread commercialization, non-chromatographic alternatives are preferable. This limitation has driven research into techniques such as membrane technology, magnetic separations, affinity precipitation, and aqueous two-phase extraction [29]. Lectins also pose practical challenges as therapeutic candidates because their stability and solubility are highly sensitive to environmental conditions. Although usually water-soluble, their behavior in solution varies with factors such as pH, ionic strength, temperature, and storage time, and they may aggregate or denature during frozen storage. Lyophilization is often used to improve shelf-life, but some lectins resist proper rehydration due to irreversible damage or inadequate stabilizing agents. As a result, careful handling—clarifying samples, verifying protein concentration, and avoiding excessive agitation—is essential to ensure reliable performance in preclinical assays [15].

## 3. Assessing the Safety of Natural Products

Due to complications associated with conventional treatments, such as drug resistance and adverse effects, natural compounds are increasingly being proposed as part of new therapeutic strategies to complement or even replace conventional medicine approaches. Furthermore, the wide range of pharmacologically active plant compounds has captured the interest of the pharmaceutical industry. These natural ingredients typically exhibit greater specificity for target cells, which can help address the growing issues of toxicity and resistance to commercially available drugs [30,31]. Additionally, natural products are generally more affordable than synthetic drugs, making them attractive alternatives [5].

The wide array of bioactive compounds produced through plant metabolism forms an integral part of plants’ defense mechanisms, enabling them to withstand various environmental stresses such as drought, salinity, nutrient deficiency, and attacks by pathogens or herbivores. While these compounds serve essential protective functions for plants, they can also pose risks to human health if misused or inadequately regulated [5,32,33,34]. Some plants, like *Ricinus communis* L., *Digitalis purpurea* L. and *Carlina gummifera* (L.), are inherently toxic due to their chemical constituents, and consuming them (or extracts derived from them) can result in poisoning [35,36,37,38]. Notably, some of these compounds (e.g., cardiac glycosides, tropane alkaloids, and takanes) can be fatal depending on the dose [38].

Indeed, any compound whose mechanism of action is not fully understood must undergo rigorous testing to establish its safety and efficacy [5]. Toxicity assessment involves studying the adverse effects of chemicals on organisms, organs (organotoxicity), or cells (cytotoxicity) [35]. Applying reliable methods to measure and predict toxicity is crucial, as unwanted effects are a leading cause of drug failure [39]. Given the chemical complexity and potential multidimensional effects of medicinal plants, their toxicological properties must be examined through multiparametric analysis, which helps identify any harmful effects [7]. Toxicological characterization can be performed using both in vitro and in vivo tests.

There are numerous cytotoxicity assays available to assess various aspects of cell health, including metabolic activity, plasma membrane integrity, changes in cell number and morphology, growth/proliferation, and cell death mechanisms [35,40]. Furthermore, more refined screening can be performed by combining advanced techniques in modern cell biology, high-resolution automated microscopy, and integrated flow cytometry. These methods allow for the simultaneous detection of multiple parameters, such as nuclear area/intensity, intracellular calcium levels, mitochondrial membrane potential, plasma membrane permeability, and cell count. This integrated approach enables the characterization of compound actions and the identification of their toxicity mechanisms, which may include mitochondrial dysfunction, oxidative stress, alterations in calcium homeostasis, and cell death.

However, in vitro toxicity tests should not replace or dispense in vivo analyses, and vice versa, as a critical factor in toxicology is the in vivo metabolism. Some substances that do not show toxicity initially can produce toxic metabolites after being exposed to liver enzymes, for example, while other substances that are toxic in vitro may have their toxicity reduced or abolished by metabolic transformations. Other factors, such as the ability of the substance to penetrate tissues and the clearance and excretion of the product, cannot be measured using cellular models [35,41].

Several well-established methods can be used to measure the in vivo toxicity of a given compound, mainly in rodents. The acute toxicity assay involves evaluating substances administered in one or more doses over a short period, not exceeding 24 h. For these purposes, the maximum dose administered to animals should not exceed 2000 mg/kg. Repeated-dose toxicity assays can be divided into subacute, subchronic, and chronic evaluations, depending on the dosage and duration of administration of the test agents. These analyses help characterize the toxicological profile of a compound. Several OECD (Organization for Economic Co-operation and Development) guidelines can be used to support the experimental design for assessment of acute [42], sub-acute [43,44] and chronic [45] toxicity, as well as reproductive toxicity [46,47] and genotoxicity [48].

In addition to assessing mortality by determining the median lethal dose (LD_50_), the experimental approach can be designed to provide information on physiological, hematological, biochemical, and histopathological alterations. The state of vital organs, especially liver and kidney functions, is crucial. The liver is involved in the metabolism of ingested compounds, while the kidneys are responsible for their excretion. Furthermore, the duration and reversibility of toxicity must be recorded and compared to the test control group.

However, toxicity testing of natural products is limited by major biological, methodological, and practical challenges. Species differences in metabolic pathways make rodent findings difficult to translate to humans [49,50]. In addition, phytochemicals can exhibit nonlinear dose–response behaviors [51]. The lack of specific biomarkers and the scarcity of pharmacokinetic data further complicate evaluation [52]. Finally, the high costs and existing regulatory gaps related to herbal products contribute to the challenges in establishing their safety.

The toxicological assessment of proteinaceous substances with pharmacological potential is especially challenging due to several factors. First, the route of administration must be carefully aligned with that used in the pharmacological study, because proteins can be unstable and/or degraded by digestive enzymes when administered orally (*per os*). In such cases, observed toxic or desired effects may not be attributable solely to the native protein, but could also result from degraded fragments [53,54]. Likewise, a lack of toxicity via the oral route does not preclude potential toxicity when administered intraperitoneally or intravenously, as enzymatic breakdown may eliminate toxic effects. Therefore, multiple routes of administration should be evaluated. Lectins, in particular, often exhibit complex and multifunctional biological effects, making it difficult to identify the primary causes of adverse effects. Consequently, multiple aspects must be studied to comprehensively characterize their toxicity. For these reasons, the study of lectin safety requires a case-by-case approach that combines in vitro, in vivo, biochemical, immunological, and histopathological methods to provide a robust toxicological assessment.

## 4. Toxicity Evaluation of Lectins

### 4.1. Assessment of In Vitro Cytotoxicity of Plant Lectins

Lectins were initially known for their toxicity due to the identification of ricin, a toxic protein present in *R. communis* seed extract [55]. Lectins toxic to healthy human cells have been described [56,57,58]. On the other hand, it has been well reported that lectins that are cytotoxic to tumor cells or pathogen cells can also be non-toxic to normal (non-tumorous or non-pathogenic) cells from human or other animals at the doses at which they exhibit their bioactivities [59,60,61,62,63].

The carbohydrate-dependent binding to specific glycan motifs on the cell surface confers this selectivity of lectins toward particular cell types. In some cases, lectin–glycan interactions can lead to direct membrane perturbation, alter ion fluxes, and cause loss of membrane integrity. Downstream effects frequently involve oxidative stress, characterized by increased ROS generation, mitochondrial dysfunction, and activation of intrinsic apoptotic pathways, including caspase activation and cytochrome c release. Additional mechanisms reported for certain lectins include modulation of MAPK and NF-κB signaling, induction of endoplasmic reticulum stress, and engagement of immune-related pathways that amplify cytotoxic or antiproliferative effects. Lectin-mediated cytotoxicity typically arises from a combination of glycan recognition, membrane-associated events, and stress-induced cell death signaling rather than from a single isolated pathway [64,65,66].

Human peripheral blood mononuclear cells (PBMCs), mesenchymal stem cells isolated from human umbilical cord, mouse splenocytes, human fibroblasts, mouse peritoneal macrophages, and Vero cell lines have been used for in vitro evaluation of natural products. Determining the degree of lectin toxicity for normal cells is important because a lack of selectivity can make their use unfeasible. Table 1 describes lectins isolated from plants that did not show cytotoxicity toward normal cells.

The lectin from *Calliandra surinamensis* Benth. leaf pinnulae (CasuL), at concentrations ranging from 1.0 to 100.0 µg/mL, did not affect the viability and cellular metabolism of PBMCs, as verified by the MTT [(4,5-dimethylthiazol-2-yl)-2,5-diphenyl tetrazolium bromide] assay, which measures mitochondrial metabolic activity. Parallelly, this lectin showed a significant cytotoxic effect against chronic myeloid leukemia cells (K562) and breast cancer cells (T47D) [71] and displayed bacteriostatic and antibiofilm activity against some mastitis isolates [75], highlighting the biological selectivity of CasuL. Lectins from *Microgramma vacciniifolia* (Langsd. & Fisch.) Copel. fronds (MvFL; 25–6.25 µg/mL) and rhizomes (MvRL; 1–100 µg/mL) were also not cytotoxic to PBMCs as indicated by apoptosis/necrosis and MTT assay, respectively. MvFL (12.5 µg/mL) was able to induce the production of cytokines (Th1) and nitric oxide, as well as activation and differentiation of T lymphocytes [61], while MvRL reduced the viability of lung mucoepidermoid carcinoma (NCI-H292) cells [72]. The *Egletes viscosa* (L.) Less. lectin (EgviL) only reduced the viability of PBMCs at the highest concentration tested (100 μg/mL), as determined by MTT assay [67]. In turn, the lectin from *Moringa oleifera* Lam. seeds (WSMoL; 1.56–100 µg/mL) did not alter the viability of PBMCs (MTT assay) after 72 h of incubation and demonstrated in vitro anti-inflammatory potential (6.25 µg/mL), reducing the production of nitric oxide and tumor necrosis factor (TNF) α by murine macrophages stimulated by lipopolysaccharide. Conversely, the coagulant lectin from *M. oleifera* seeds (cMoL; 1.56–100 µg/mL) was potentially cytotoxic to PBMCs, as determined by IC_50_ = 11.72 µg/mL (MTT assay), indicating potential safety concerns [57].

Another lectin that did not exhibit cytotoxicity toward PBMCs was that isolated from the fruit exudate of *Praecitullus fistulosus* (Stocks) Pangalo (PfL); however, it demonstrated a cytotoxic effect against the colon cancer cell line (HT29), where the treated cells showed apoptotic characteristics, including membrane blebbing, degraded nuclear membrane, and condensed chromosomes. Western blot results showed that PfL treatment caused the upregulation of Bax protein and downregulation of Bcl-2. A caspase-3 inhibitor assay confirmed the involvement of caspase-3 in DNA fragmentation [76]. The lectin isolated from phloem exudates of *P. fistulosus* (PfLP), even at a concentration of 1000 µg/mL, also did not demonstrate cytotoxicity to PBMCs evaluated by MTT assay but presented significant cytotoxic potential against tumor lines of HT29, HeLa, MCF-7, and K562 [65]. Finally, the lectin from *Punica granatum* L. sarcotesta (PgTeL; 1–100 µg/mL) did not show cytotoxicity for PBMCs (apoptosis/necrosis assessment) but was capable of inducing damage to the cell wall of *Candida albicans* and *Candida krusei* [77] and showed antibacterial activity against standard and antibiotic-resistant isolates of *Escherichia coli* [78], *Staphylococcus aureus* [79], and *Pseudomonas aeruginosa* [80]. Together, the data presented above show that multiple plant lectins exhibit no toxicity to PBMCs while displaying diverse bioactivities, including anticancer, immunomodulatory, anti-inflammatory, and antimicrobial effects, highlighting their potential to target harmful cells without compromising the patient’s immune function.

Splenocytes from BALB/c mice treated with CasuL (12.5 µg/mL) showed a small increase in the level of cytosolic reactive oxygen species (ROS), but without changes in the levels of mitochondrial ROS, in addition to stimulating cell proliferation and production of IL-2 cytokines and TNF-α. This showed that the lectin could activate immune cells without causing their death, as there was no change in cell viability (i.e., absence of apoptosis or necrosis) [62]. Mice splenocytes treated with the lectin from *Schinus terebinthifolia* Raddi. leaves (SteLL) at 12.5 µg/mL did not suffer interference in the levels of ROS, cytosolic [Ca^+2^], or mitochondrial membrane potential, nor did they undergo apoptosis or necrosis. Interestingly, these splenocytes were immunomodulated by the lectin, which induced the production of Th1 and Th17 cytokines [63]. PgTeL also did not affect the viability of mouse splenocytes (3.12–200 μg/mL) [81]. Thus, these lectins can modulate immune cell function without compromising cell viability, highlighting their potential as safe immunomodulatory agents.

In an evaluation with human fibroblast cell line, cMoL (1.5–16 µM) did not change the viability of these cells after 48 h, while it reduced the viability of melanoma tumor cells (B16-F10; IC_50_ = 9.72 µM). Apoptosis, necrosis and morphological changes were observed in the tumor cells [82]. Importantly, although cMoL was potentially cytotoxic to PBMCs [57], these data show that it is not broadly toxic, as it did not harm fibroblasts. The lectin isolated from *Abelmoschus esculentus* (L.) Moench seeds (AEL; 0.1–0.0125 mg/mL) did not present cytotoxic effects on human fibroblasts exposed for 24, 48, and 72 h. However, it caused a cytotoxic effect against the cancer line MCF-7 at a concentration of 0.1 mg/mL after 72 h of incubation. The treated cells showed apoptotic characteristics, as analyzed by flow cytometry and caspase-3 and -9 mRNA, as well as p21 gene expression [73].

Souza et al. [74] evaluated the cytotoxic effects of lectins isolated from the latex of *Synadenium carinatum* Boiss. (ScLL; 0.06–50 µg/mL) and the seeds of *Artocarpus heterophyllus* Lam. (ArtinM; 0.00004–1.0 µg/mL) in macrophages isolated from the bone marrow of mice. After 24 h of incubation, viability rates (MTT assay) greater than 80% were observed. Furthermore, these lectins were tested in mice infected with the *Toxoplasma gondii* strain (ME49) and showed immunotherapeutic potential in controlling this infection.

Hemolytic activity, which involves the destruction of erythrocytes, is frequently studied in various contexts, such as infections, and transfusion reactions. New compounds can be tested to see if they cause hemolysis, helping to assess the safety of their use in healthy cells. For example, the lectin isolated from *Portulaca elatior* Mart. ex Rohrb. leaves (PeLL; 2.34–300 µg/mL) and PgTeL (3.12–200 μg/mL) did not affect human erythrocyte viability [81,83].

### 4.2. Assessment of In Vivo Toxicity of Plant Lectins

The toxicity of lectins has been assessed in rodents following oral, intraperitoneal, and intravenous administration, using survival rates as well as biochemical, hematological, and histopathological parameters. The data on the toxicity of plant lectins in in vivo assays are diverse. Some lectins do not exert any adverse effects via any administration route (oral, intraperitoneal, intravenous) in animals. Others induce toxicity signs only when repeatedly administered, while some can be considerably harmful even with a single dose. Below, we discuss the articles that explore the safety of plant lectins, followed by those that report the toxic effects of these proteins.

Acute toxicity studies with lectins assessed their toxic effects after a single exposure, being the animals usually monitored for up to 14 days. A lectin isolated from the leaves of *Bauhinia monandra* Kurz (BmoLL) was evaluated for acute oral toxicity at 300 and 2000 mg/kg and considered to be non-toxic, as it did not cause mortality or alter the body weight of Swiss mice (*n* = 3 per group) [84]. The safety of BmoLL for non-target organisms has been carefully investigated [85], and its relevance stems from the fact that BmoLL exhibits anti-inflammatory and antinociceptive activities in murine models [86], while also functioning as an insecticidal agent against stored-product pests [87]. In this last case, ensuring its safety is therefore essential for defining potential application strategies, especially in the context of grain protection for animal and human consumption.

The lectin from *Musa acuminata* Colla phloem exudates (MAL) was administered orally (2000 mg/kg) to female Swiss albino mice (*n* = 3 per group), and the results showed no suggestive effects of acute toxicity (such as changes in motor and feeding activities, behavioral changes, or physical appearance), and there were no animal deaths. Additionally, treatment with MAL showed anti-cancer potential, inhibiting the growth of Ehrlich Ascites Carcinoma (EAC), suppressing neoangiogenesis, and increasing the survival rate of treated animals [88].

However, these studies with BmoLL and MAL did not evaluate parameters such as hematological profiles, target-organ histopathology, and blood biomarkers of metabolic homeostasis and organ function, so that if such alterations occurred, they were not detected, despite the absence of mortality or behavioral changes.

PeLL was evaluated for acute oral toxicity (5 and 10 mg/kg), and no signs of toxicity were observed in treated Swiss mice (*n* = 3 per group) compared to controls that received only vehicle. This was confirmed by daily assessments of body weight, water intake, food consumption, and hematological parameters (including red and white blood cell counts and red blood cell indices) and biochemical markers (liver and kidney function tests). In the same study, PeLL demonstrated antimicrobial potential against bacteria and fungi of the genera *Pectobacterium* and *Candida*, respectively, encouraging further research for the development of products and strategies utilizing this plant lectin as an antimicrobial agent [83]. Intraperitoneal administration of *M. acuminata* pseudostem lectin (MALP; 10 mg/kg) in Swiss mice (*n* = 5 per group) showed no acute toxicity, as there were no deaths, no adverse effects, and no changes in blood parameters, including alkaline phosphatase, creatinine, urea, erythrocytes, and leukocytes; MALP was found to inhibit EAC tumor development [89]. However, histopathological assessments were not performed in these studies with PeLL and MALP.

PgTeL was evaluated for acute toxicity and genotoxicity when intraperitoneally (100 mg/kg) administered in mice (*n* = 5 per group). It did not cause signs of toxicity such as piloerection, changes in fecal appearance, sensitivity to sound or touch, altered mobility, or aggressive behavior, nor did it affect body weight, water, or food intake. Additionally, biochemical parameters (including liver and kidney function tests and lipid profile) and hematological parameters (including red and white blood cell counts, red blood cell indices, and differential leukocyte counts), as well as histopathological studies of liver, kidney and spleen, were evaluated. A decrease in triglycerides, low-density lipoprotein (LDL), and very low-density lipoprotein (VLDL) levels was observed, which suggests potential for future studies on PgTeL’s hypolipidemic effects [81].

SteLL was evaluated for acute toxicity and genotoxicity in Swiss mice (100 mg/kg; *n* = 3 per group) when administered by oral or intraperitoneal route. It did not cause signs of toxicity, and no changes were found in hematological and biochemical parameters (similar to those evaluated for PgTeL), or histopathological analyses of the liver, kidney and spleen. In addition, the authors evaluated possible gastric toxicity, but no histological alterations in the stomach were observed [90]. These preclinical safety data are highly relevant, as studies with the lectin SteLL have revealed a range of promising therapeutic activities, including antitumor [91], antinociceptive [90], anti-inflammatory [90], and anxiolytic–antidepressant effects [92]. Given this therapeutic potential, it is essential to intensify safety investigations under subacute and chronic administration regimens of SteLL to support its future development and clinical translation.

In contrast to the lectins discussed so far, some members of this protein class have demonstrated acute toxicity in rodents, in certain cases with effects that vary according to the route of administration. Lectin isolated from *Tetracarpidium conophorum* (Müll.Arg.) Hutch. & Dalziel seeds (TCL) was evaluated for acute oral toxicity (500, 750, 1000, 1500, 2000, and 2500 mg/kg) in Wistar albino mice (*n* = 6 per group). It did not cause observable body weight changes, mortality, or signs of toxicity (such as convulsions, hypoactivity, salivation, ataxia, weakness, and respiratory depression). However, when administered intraperitoneally (10, 20, 40, 80, 160, 320, and 600 mg/kg), remarkable toxicity was observed with an LD_50_ of 50 mg/kg. These animals showed immediate hyperactivity after administration, but were soon followed by a decrease in movements and respiratory rate. In addition, the authors described the occurrence of convulsions preceding death [93].

The lectin isolated from *Bixa orellana* L. leaves (BoLL), at 100 mg/kg (both intraperitoneally or *per os*), did not promote death of intoxicating signals in Swiss mice (*n* = 5 per group), and no alterations were found in hematological and biochemical analyses. However, histopathological changes were observed in the kidneys (small vacuolation, increased capsular space with decreased glomerular tuft, and small lymphocytic infiltrates), liver (lymphocytic infiltrates, small vacuolated cells, and a fibrosis process), and spleen (disorganization of lymphoid tissue, lymphocytic infiltrates, small granulomas, and an increased number of macrophages) in animals treated with a single dose of BoLL via both routes [94]. These results highlight the importance of assessing histopathological changes, not just mortality, to reveal subtle organ effects and obtain a more complete evaluation of BoLL’s safety.

The acute toxicity of WSMoL (100 and 200 mg/kg) was evaluated intraperitoneally in Swiss mice (*n* = 5 per group). At 100 mg/kg, reduced food and water consumption, as well as weight loss, were observed in Swiss mice, but no signs of toxicity were identified in hematological (red cell counting and indices, and total and differential leukocyte counts), biochemical (liver and kidney function markers), or histopathological analyses of liver, kidney, and spleen. However, WSMoL at 200 mg/kg caused 40% mortality by the second day after administration and 60% mortality by the third day. Soon after administration, the mice that received this dose showed decreased ambulation, constipation, and abdominal spasms. Due to these effects, the authors did not perform hematological, biochemical, and histopathological analysis of animals treated with 200 mg/kg and concluded that it is not safe to use WSMoL intraperitoneally in doses greater than 100 mg/kg. They also showed the in vivo antitumor activity of the lectin at safe doses (up to 10 mg/kg i.p.) [95]. The risk assessment of WSMoL is particularly important given the widespread use of *M. oleifera* seeds in water treatment and the need to carefully control dosing when exploring its pharmacological potential. This is especially relevant because WSMoL has demonstrated antihypertensive action [96], as well as anxiolytic and antidepressant activities [97], all at doses shown to be safe.

When evaluating the acute oral toxicity of *Ziziphus mauritiana* Lam. seed lectin (ZMSL) at doses ranging from 2 to 16,000 μg/kg in Wistar albino rats (*n* = 6 per group), behavioral changes such as sluggish movement, decreased appetite, and sticky liquid stool were observed in animals that received 8000 μg/kg dose. Mortality was noted in animals starting at the dose of 1000 μg/kg. In this context, the authors assessed the pharmacological potential of ZMSL at 800 μg/kg and found that it fully prevented anaphylactic shock and the Arthus reaction in vivo, demonstrating its pronounced antiallergic activity [98].

Acute toxicity assessment of the lectin from *Mucuna pruriens* (L.) DC. seeds (MpLec) was using a less commonly studied route of administration: the lectin was intravenously administered (5 and 10 mg/kg) to Swiss mice (*n* = 10 per group), revealing no signs of toxicity. In the same study, MpLec demonstrated gastroprotective and antioxidant actions in an experimental model of ethanol-induced gastropathy [99].

Repeated-dose assays to check the toxicity of lectins have also been performed. PfLP, when administered (10 mg/kg) intraperitoneally three times on alternate days to Swiss mice (*n* = 6 per group), did not show toxicity based on absence of deaths and no changes in hematological parameters (red and white blood cell counts, and hemoglobin) and biochemical markers of liver and renal function; the lectin was also able to inhibit tumor growth and progression in EAC-bearing mice, in addition to suppressing tumor angiogenesis [68]. Intraperitoneal administration of PfL (10 mg/kg) to EAC-bearing Swiss mice (*n* = 5) on alternate days for 55 days did not cause toxicity, as proven by biochemical parameters (alanine aminotransferase, aspartate aminotransferase and creatinine) and histopathological analysis of the liver, kidney, and spleen. PfL treatment also demonstrated anticancer potential, improving survival in comparison with untreated EAC-bearing animals [76].

Treatment with the lectin from *Lonchocarpus araripensis* Benth seeds (LAL; 1 mg/kg) for seven consecutive days showed no signs of toxicity when administered intravenously in Wistar rats (*n* = 7 per group), as indicated by evaluation of body mass, organs wet weight, hematological (total and differential leukocyte counting), biochemical parameters (liver and kidney function markers), and macroscopic observation of spleen, kidney, liver, heart, and stomach. The microscopic analysis was not achieved. The study demonstrated that the lectin has anti-inflammatory effects in models of acute inflammation (paw edema and peritonitis induced by carrageenan) by reducing leukocyte migration [100].

Repeated intraperitoneal injections (every 7 days for 4 weeks) of lectin isolated from *Echinacea purpurea* (L.) Moench roots (LysM) at 250 µg/kg were administered in BALB/c mice (*n* = 15 per group) and nephrotoxicity was specifically evaluated. The data revealed renal changes such as glomerular vacuolation and tubular necrosis, protein cylinder deposits indicating a disrupted tubular secretion, and an increase in glomerular diameter, possibly due to increased cellular hypertrophy and hyperplasia, as well as increased hyperfiltration [101]. These findings indicate the need for careful evaluation of the pharmacological potential of this lectin.

Finally, a lectin isolated from *Phaseolus acutifolius* A.Gray seeds (TBLF; 50 mg/kg every third day for 6 weeks), in a subchronic oral toxicity study, caused antinutritional effects, including a decrease in food intake and body weight in Sprague-Dawley rats (*n* = 4 per group) during the first weeks. Additionally, blood analysis within the first 24 h after administration showed an allergic response (high level of eosinophils), which disappeared after four weeks of treatment [102]. Alatorre-Cruz and collaborators evaluated TBLF (50 mg/kg) in Sprague-Dawley rats (*n* = 12 per group) for six weeks, followed by two weeks of recovery. However, the recovery period was not sufficient to reduce the pancreatic hypertrophy caused by compensation for intestinal digestion dysfunction [103]. Subsequently, a study assessing the adverse effects of TBLF on the rat digestive tract reported small intestinal atrophy, villus shortening, crypt hyperplasia, increased mucus production, enhanced intestinal permeability, and reduced apparent ileal digestibility, although many of these changes were partially reversed after six weeks of recovery [104]. These findings are particularly relevant given TBLF’s potential as an anticancer agent against colon cancer cells [105], emphasizing the importance of balancing therapeutic efficacy with gastrointestinal safety. Table 2 provides a summary of the effects of the toxic lectins discussed in this review.

## 5. Limitations, Knowledge Gaps and Future Directions

The study of lectin toxicity has presented some methodological issues. Although many studies have adhered to the core principles of OECD protocols, the doses evaluated have not been standardized across investigations. Also, some lectins do not exhibit obvious toxic effects in terms of mortality, body weight, or behavior; others show significant toxicity depending on the route of administration or dose, highlighting the need for assessment under multiple experimental conditions. Many studies evaluate only one route of administration, usually oral, which does not guarantee that these proteins are also safe when administered intraperitoneally or intravenously.

**Figure 3:**
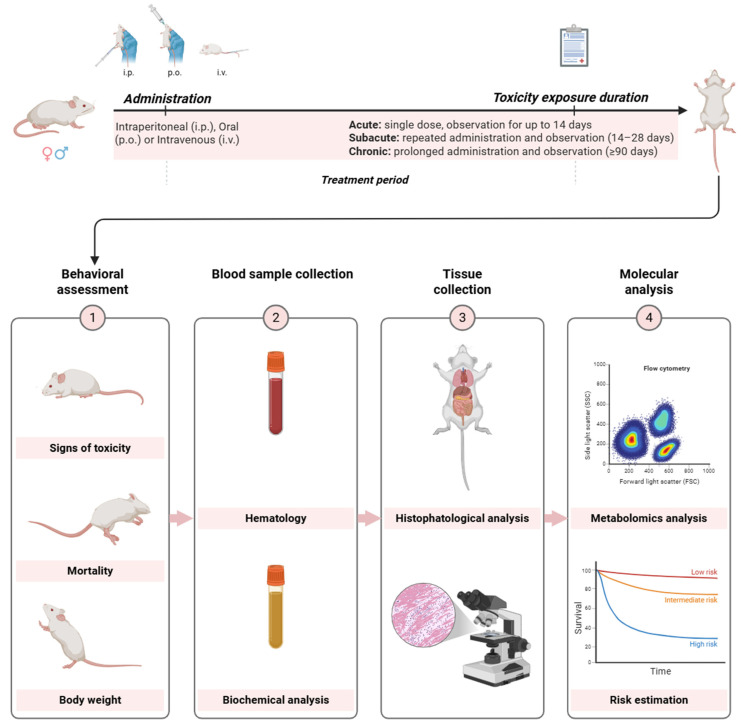
A suggested framework to assess toxicity of lectins in rodent models. Toxicity studies begin with the administration of the test substance, which can occur via different routes. The exposure period varies according to the study design. Throughout the treatment period, toxicity is assessed through four major stages. Animals are monitored for general signs of toxicity, alterations in posture or movement, changes in grooming behavior, and other clinical symptoms. Mortality is recorded daily, and body weight is tracked as an important indicator of general health and systemic toxicity. Blood sample collection is performed to check hematological and biochemical parameters. At the end of the exposure period, animals are euthanized for macroscopic examination and organ harvesting. Tissues such as the liver, kidneys, heart, lungs, and spleen are collected and processed for histopathological analysis. Additional molecular endpoints can be measured using techniques such as flow cytometry, proteomics, or metabolomics. Created using BioRender.

Another critical point is that many studies do not investigate more detailed parameters, such as hematological profiles, biochemical biomarkers, metabolic function, and histology of target organs. Thus, subclinical changes may go unnoticed, even in the absence of death or behavioral signs. There is evidence that some lectins can cause histological changes in key organs such as the liver, kidneys, or spleen without apparent clinical effects. In this context, Figure 3 presents a comprehensive and structured framework intended to guide the systematic assessment of lectin toxicity, ensuring that critical parameters relevant to safety evaluation are rigorously examined.

Studies with repeated or subchronic administration demonstrate additional effects that would not be detected in acute tests. Some reports indicate that repeated doses may cause mild to moderate physiological or metabolic changes, temporary antinutritional effects, or compensatory alterations in organs related to digestion, reinforcing the importance of prolonged monitoring. In this sense, omics-based toxicology approaches and computational modeling can help generate more detailed and precise insights into lectin safety.

These findings highlight the need for more extensive and comprehensive toxicity studies, involving multiple routes of administration, varied doses, hematological, biochemical, and histological analyses, as well as subchronic and chronic toxicity evaluations. Moreover, the balance between safety and potential therapeutic effects should be considered, as some lectins exhibit anticancer, anti-inflammatory, antimicrobial, gastroprotective, or hypolipidemic properties at safe doses. Thus, the safety assessment of lectins should not be limited to mortality or behavioral observations. More detailed and long-term studies are essential to understand the full toxicity profile, identify subclinical effects, and determine the feasibility of therapeutic applications of these proteins. In addition, genotoxicity has not been assessed for most of the lectins, and no study on reproductive safety has been conducted.

## 6. Conclusions

The data reviewed in this paper on the toxicity of lectins indicate that several of these proteins have not been shown to be potentially toxic to human and animal cells, nor when administered to rodents orally, intraperitoneally, or intravenously, based on survival, biochemical, hematological, and histopathological data. However, some lectins have been shown to be dangerous, depending on the administration route, dose, and treatment duration, causing behavioral alterations, antinutritional effects, hepatotoxicity, nephrotoxicity, pancreatic hypertrophy, allergic responses, and even death. Therefore, it is crucial to emphasize the importance of investigating toxicity to provide information on possible adverse reactions and ensure safety before their commercialization as herbal medicines.

## Figures and Tables

**Table 1:** Non-cytotoxic plant lectins to non-tumorous and non-pathogenic cells in vitro and their potential applications.

Plant Tissue (Plant)	Lectin	Cell Type	Pharmacological Potential	References
Floral capitula (*Egletes viscosa*) Fronds (*Microgramma vacciniifolia*)	EgviL	PBMCs	Cytotoxic against tumor cells	[67]
	MvFL	PBMCs	Immunomodulator	[61]
Fruits (*Praecitullus fistulosus*)	PfL	PBMCs	Antitumor	[68]
Inflorescences (*Alpinia purpurata*)	ApuL	PBMCs, MSCs	Immunomodulator; cytotoxic against tumor cells	[69,70]
Leaf pinnulae (*Calliandra surinamensis*)	CasuL	PBMCs, Splenocytes	Antimicrobial, cytotoxic against tumor cells; immunomodulator	[62,71]
Leaves (*Schinus terenbithifolia*)	SteLL	Splenocytes; MSCs	Immunomodulator; cytotoxic against tumor cells	[63,69]
Rhizomes (*Microgramma vacciniifolia*)	MvRL	PBMCs	Cytotoxic against tumor cells	[72]
Seeds (*Abelmoschus esculentus*)	AEL	Fibroblasts	Cytotoxic against tumor cells	[73]
Seeds (*Artocarpus heterophyllus*)	ArtinM	Bone marrow macrophages	Immunomodulator	[74]
Seeds (*Canavalia brasiliensis*)	ConBr	Peritoneal macrophages	Immunomodulator	[60]
Seeds (*Cratylia argentea*)	CFL	Peritoneal macrophages	Immunomodulator	[60]
Seeds (*Moringa oleifera*)	WSMoL	PBMCs	Anti-inflammatory	[57]

PBMCs: peripheral blood mononuclear cells. MSCs: mesenchymal stem cells.

**Table 2:** Comparative toxicity of plant-derived lectins across animal models and exposure routes.

Plant Tissue (Lectin)	Animal Model	Route and Regimen	Endpoint (Dose)	References
*Bixa orellana* leaves (BoLL)	Mice	Intraperitoneal and oral (single dose)	Kidney, spleen and liver damage (100 mg/kg)	[94]
*Echinacea purpurea* roots (LysM)	Mice	Intraperitoneal (repeated doses)	Kidney damage (250 µg/kg)	[101]
*Moringa oleifera* seeds (WSMoL)	Mice	Intraperitoneal (single dose)	Mortality (200 mg/kg)	[95]
*Phaseolus acutifolius* seeds (TBLF)	Rats	Oral (repeated doses)	Allergic response; pancreatic hypertrophy; intestinal lesions (50 mg/kg)	[103,104,105]
*Tetracarpidium conophorum* seeds (TCL)	Mice	Intraperitoneal (single dose)	Mortality (LD_50_: 50 mg/kg)	[93]
*Zizyphus mauritiana* seed (ZMSL)	Rats	Oral (single dose)	Mortality (1000 μg/kg)	[98]

## Data Availability

No new data were created or analyzed in this study. Data sharing is not applicable to this article.
